# Would Any Other Treatment Have Produced a Cure?

**Published:** 1877-04

**Authors:** J. M. Porter


### WOULD ANY OTHER TREATMENT HAVE PRODUCED A CURE?

BY J. M. PORTER D. D. S.

In the early part of the summer of 1876, I was called upon by a young gentleman to insert a number of gold fillings. Among the operations made at that time was a small gold filling in the mesial surface of the left superior lateral incisor. Everything remained comfortable until January 9th, 1877, when the young man returned and said he had pain in the tooth to which I have just referred, and upon making an examination I discovered a small cavity on the distal surface, which I immediately filled. He returned on January 15th and said he still suffered considerable pain. I made a careful examination of the surrounding tissues, but could not find anything to justify me in concluding that the pulp had died; because he did not complain of pain on percussion, nor did the tissues look inflamed above and around the tooth. Failing in finding any other cause for the trouble, I concluded the pulp must be dead, and I drilled a small opening through the lingual surface into the pulp cavity, and found that the pulp had evidently been dead for sometime. I washed out and filled the nerve canal, and the cavity I had made in reaching it, with gold. I then supposed I had found the cause of the trouble and so assured my patient. But to my chagrin the patient returned on January 19th, and said his tooth still gave him pain and wished me to extract it, and said "he was getting tired of the thing." I secured from him the privilege of extracting and replanting, having then concluded I could definitely decide as to the cause of his trouble by extracting only. I extracted the tooth and found upon the apex of the root a slightly exostosed condition, and the surface of the end of the root was quite rough; I cut off the enlarged portion and polished the end of the root and replaced the tooth, without giving either the tooth or socket any further treatment. In less than two days the tooth was perfectly comfortable, and has remained so ever since. It is now as firm as ever, and as useful. The patient has never lost a tooth, and since the "siege is over" we are both very much gratified to think that he did not lose the one in question. This was a case not easily diagnosed, as the "sensations" were very deceptive. I have reported it in order to encourage others to "hold the fort," even though opposed by their patients, and confronted by new obstacles in their efforts to preserve the natural teeth.

